# Supplementary material for: Identification of photoperiod-induced specific miRNAs in the adrenal glands of Sunite sheep (Ovis aries)
Source: Front Vet Sci. 2022 Jul 22;9:888207. doi: 10.3389/fvets.2022.888207 (PMC9354845; doi:10.3389/fvets.2022.888207)
Supplement: Supplementary file 1 [file Data_Sheet_1.ZIP › Supplementary file/miRDeep2 file.zip!/miRDeep2 file/Novel_Structure/SPLP42A2/Novel_550.pdf]

Provisional ID	:	NC_019468.2_22854
Score total	:	95.2
Score for star read(s)	:	3.9
Score for read counts	:	87.3
Score for mfe	:	2.4
Score for randfold	:	1.6
Score for cons. seed	:	
Total read count	:	183
Mature read count	:	182
Loop read count	:	0
Star read count	:	1

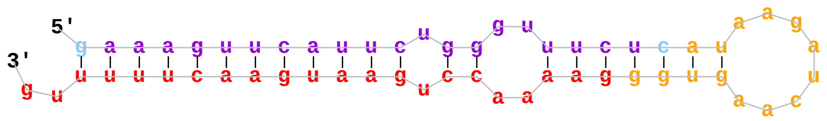

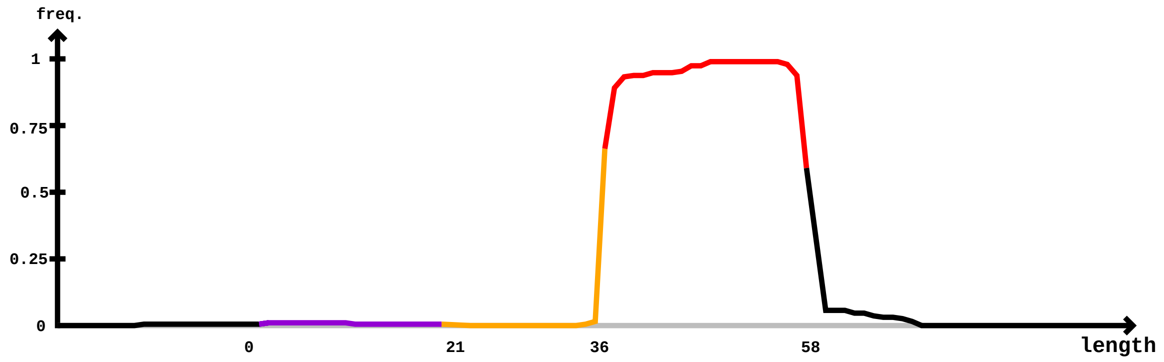


```
                     Star                              Mature
5'- ugaucuauuggguuggccagaaaguucauucuggguuucucauaagaucaaguggggaaaaccugaaugaacuuuuuggccaacccaauacuuccugucucuggaucgccc   -3'   obs
    ugaucuauuggguuggccagaaaguucauucuggguuucucauaagaucaaguggggaaaaccugaaugaacuuuuuggccaacccaauacuuccugucucuggaucgccc         exp
    .....((((((((((((((((((((((((((.((..(((((((........)))))))..)).))))))))))))))))))))))))))..(((.......)))......   reads   mm   sample
    .......uuggguuggccagaaaguucau.................................................................................   1       0    seq
    ....................aaaguucauucuggguuucu......................................................................   1       0    seq
    .....................................................gggaaaaccugaaugaacuuuu...................................   1       0    seq
    ......................................................ggaaaaccugaaugaacCuu....................................   1       1    seq
    ......................................................ggaaaaccugaaugaacCuuuu..................................   1       1    seq
    .......................................................gaaaaccugaaugaacCuuu...................................   6       1    seq
    .......................................................gaaaaccugaaugaacCuuuu..................................   43      1    seq
    .......................................................gGaaaccugaaugaacuuuuu..................................   1       1    seq
    .......................................................Caaaaccugaaugaacuuuuu..................................   1       1    seq
    .......................................................gGaaaccugaaugaacuuuuug.................................   2       1    seq
    .......................................................gaaaaccugaaugaacCuuuug.................................   69      1    seq
    .......................................................gaaaaccugaaugaacCuuuugg................................   2       1    seq
    .......................................................gaaaaccugaaugaacuuuuugU................................   1       1    seq
    ........................................................aaaaccAgaaugaacuuu....................................   1       1    seq
    ........................................................aaaaccAgaaugaacuuuu...................................   1       1    seq
    ........................................................aaaaccAgaaugaacuuuuu..................................   7       1    seq
    ........................................................Uaaaccugaaugaacuuuuu..................................   1       1    seq
    ........................................................aaaaccugaaugaacuuuuu..................................   13      0    seq
    ........................................................aaaaccugaaugaacuuuuuA.................................   1       1    seq
    ........................................................Gaaaccugaaugaacuuuuug.................................   2       1    seq
    ........................................................aaaGccugaaugaacuuuuug.................................   1       1    seq
    ........................................................aaaaccGgaaugaacuuuuugg................................   1       1    seq
    ........................................................aaaacUugaaugaacuuuuugg................................   1       1    seq
    ........................................................aaaaccugaaugaacCuuuugg................................   7       1    seq
    ........................................................aaaaccugaaugaGcuuuuugg................................   1       1    seq
    ........................................................aaaaccugaaugaacuuuuugU................................   7       1    seq
    .........................................................aaaccugaaugaacuuuuug.................................   1       0    seq
    .........................................................aaaccAgaaugaacuuuuug.................................   1       1    seq
    .........................................................aaaccugaGugaacuuuuugg................................   1       1    seq
    .........................................................aaaccugaaugaacuuuuugU................................   3       1    seq
    .........................................................aaaccugaauAaacuuuuugg................................   1       1    seq
    .........................................................Uaaccugaaugaacuuuuugg................................   1       1    seq
    ..........................................................aaccugaaCgaacuuuuuggcca.............................   1       1    seq
```

Star Mature

```
ugaucuauuggguuggccagaaaguucauucuggguuucucauaagaucaagugggaaaaccugaaugaacuuuuuggccaacccaauacuuccugucucuggaucgccc
............................................................ccugaaugaacuuuuuggccU.............................    1    1    seq
............................................................ccugaaugaacuuuuuggccaaU...........................    1    1    seq
...............................................................gaaugaacuuuuuggccaaccca........................    1    0    seq
................................................................aaugaacuuuuuggccaac...........................    1    0    seq
................................................................aaugaacuuuuuggccaacG..........................    1    1    seq
................................................................aaugaacuuuuuggccaacccaa.......................    1    0    seq
................................................................aaugaacuuuuuggccaacccaUu......................    1    1    seq
..................................................................ugaacuuuuugCccaacccaa.......................    1    1    seq
..................................................................ugaacuuuuuggccaacccaGu......................    2    1    seq
```
